# Astragalus membranaceus Inhibits Inflammation via Phospho-P38 Mitogen-Activated Protein Kinase (MAPK) and Nuclear Factor (NF)-κB Pathways in Advanced Glycation End Product-Stimulated Macrophages

**DOI:** 10.3390/ijms13078379

**Published:** 2012-07-05

**Authors:** Qiaojing Qin, Jianying Niu, Zhaoxia Wang, Wangjie Xu, Zhongdong Qiao, Yong Gu

**Affiliations:** 1 Department of Nephrology, Shanghai Fifth People’s Hospital, Fudan University, Shanghai 200240, China; E-Mails: qinqiaojing@sohu.com (Q.Q.); njyphd2008@yahoo.com.cn (J.N.); 2 School of Life Science and Biotechnology, Shanghai Jiaotong University, Shanghai 200240, China; E-Mails: zhaoxiaw@sjtu.edu.cn (Z.W.); hover_xwj@sjtu.edu.cn (W.X.); zdqiao@sjtu.edu.cn (Z.Q.); 3 Department of Nephrology, Huashan Hospital, Fudan University, Shanghai 200240, China

**Keywords:** *Astragalus membranaceus*, advanced glycation end products, macrophage, inflammation, diabetes

## Abstract

Advanced glycation end products (AGEs) and inflammation contribute to the development of diabetic complications. *Astragalus membranaceus* has properties of immunological regulation in many diseases. The aim of this study was to determine the function of *A. membranaceus* extract (AME) on the AGE-induced inflammatory response in Ana-1 macrophages. The viability of cells treated with AME or AGEs was evaluated with the MTT [3-(4,5-dimethyl-2-thiazolyl)-2,5-diphenyl-2*H*-tetrazolium bromide] method. The secretion and mRNA levels of IL-1β and TNF-α were measured by ELISA and RT-PCR, respectively. The activity of NF-κB was assayed by EMSA. The phosphorylation of p38 MAPK was assessed by western blotting. The results showed that AME was not toxic to macrophages. The treatment of macrophages with AME effectively inhibited AGE-induced IL-1β and TNF-α secretion and mRNA expression in macrophages. These effects may be mediated by p38 MAPK and the NF-κB pathway. The results suggest that AME can inhibit AGE-induced inflammatory cytokine production to down-regulate macrophage-mediated inflammation via p38 MAPK and NF-κB signaling pathways and indicate that AME could be an immunoregulatory agent against AGE-induced inflammation in diabetes.

## 1. Introduction

A rapidly growing number of clinical and experimental data support the concept that diabetes is a chronic inflammatory disease [1,2]. Macrophages are the major immune cells infiltrating vascular tissue in diabetes. Evidence has shown that the monocyte chemo-attractant protein-1 (MCP-1)- and the C-chemokine receptor (CCR1)-dependent recruitment of macrophages contribute to the development of diabetic vascular complications, e.g., renal injury [3–6]. After long-term exposure to a hyperglycemic milieu, proteins in the blood and tissue eventually give rise to the irreversible formation of advanced glycation end products (AGEs). AGE accumulation and macrophages have been implicated in the development and progression of diabetic complications [7].

AGEs activate monocytes/macrophages to produce excess inflammatory cytokines, such as interleukin-1 beta (IL-1β) and tumor necrosis factor-alpha (TNF-α) [8,9]. IL-1β is an important component in the initiation and enhancement of inflammatory responses. TNF-α mediates inflammatory tissue injury [10–12]. TNF-α and IL-1β are associated with diabetic complications [13,14]. Therefore, substances that can inhibit the inappropriate production of TNF-α and IL-1β in AGE-stimulated macrophages could be considered as potential anti-inflammatory agents.

Immunosuppressants may prevent chronic inflammation, but they have been found to have a high infectious rate in diabetic patients. Recently, *Astragalus membranaceus*, a Chinese herb, has attracted attention as an immunomodulator that can reduce the production of TNF-α and IL-1β in lipopolysaccharide-stimulated macrophages [15].

Based on these previous reports, we investigated the effects of *A. membranaceus* extract (AME) on AGE-induced inflammation involving TNF-α and IL-1β via phospho-P38 mitogen-activated protein kinase (MAPK) and nuclear factor (NF)-κB pathways in macrophages.

## 2. Results and Discussion

### 2.1. Effects of AGEs and AME on Cell Viability

**Figure 1:**
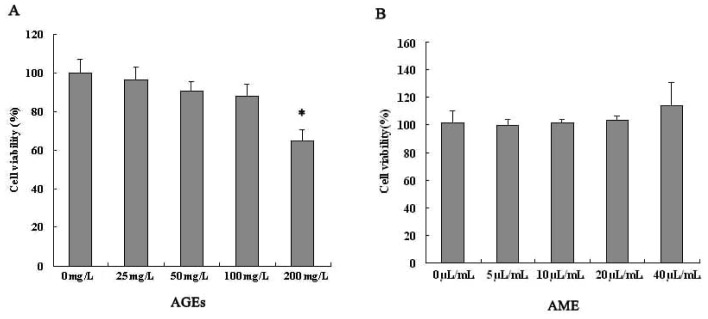
Viability analysis of Ana-1 macrophages after treatment with advanced glycation end products (AGEs) (**A**) or *Astragalus membranaceus* extract (AME) (**B**). Cells (5 × 10^4^) were treated with AME (5–40 μL/mL) or AGEs (25–200 mg/L) for 24 h. Cell viability was assessed with an MTT [3-(4,5-dimethyl-2-thiazolyl)-2,5-diphenyl-2*H*-tetrazolium bromide] assay. The results represent the mean of six culture wells (mean ± SEM). * *p* < 0.05 for 200 mg/L AGEs *vs*. control (0 mg/L). All of the experiments were performed independently in triplicate.

The treatment of Ana-1 macrophages with different concentrations of AME (5–40 μL/mL) for 24 h did not significantly affect cell viability. We found no toxicity of AME to macrophages (Figure 1B). Meanwhile, AGEs (25–200 mg/L) decreased macrophage viability in a concentration-dependent manner at 24 h (Figure 1A).

Based on these results and previous reports, we chose the maximum concentration of AME (40 μL/mL) and AGEs (100 mg/L) that did not significantly affect Ana-1 macrophage viability at 24 h for subsequent experiments to determine underlying mechanisms.

### 2.2. Effects of AME and AGEs on the mRNA Levels and Secretion of IL-1β and TNF-α in Macrophages

Diabetic complications are a leading cause of acquired blindness, end-stage renal failure and accelerated atherosclerosis. Accumulating evidence has shown that AGEs and inflammation are associated with vascular complications of diabetes [16,17]. As major effector cells of the immune response, activated macrophages produce a wide spectrum of inflammatory cytokines, such as TNF-α and IL-1β, to enhance the inflammatory response in diabetes [11,18,19].

*A. membranaceus*, a traditional Chinese herb, significantly inhibits the lipopolysaccharide-induced production of TNF-α, IL-6, IL-10, and IL-12 in macrophages [20]. Although the immunoregulatory functions of *A. membranaceus* have been demonstrated in many diseases, nothing is known about the effect of *A. membranaceus* on AGE-activated macrophages. Therefore, we examined the effects of AME on the expression of the inflammatory cytokines IL-1β and TNF-α in AGE-stimulated macrophages.

**Figure 2:**
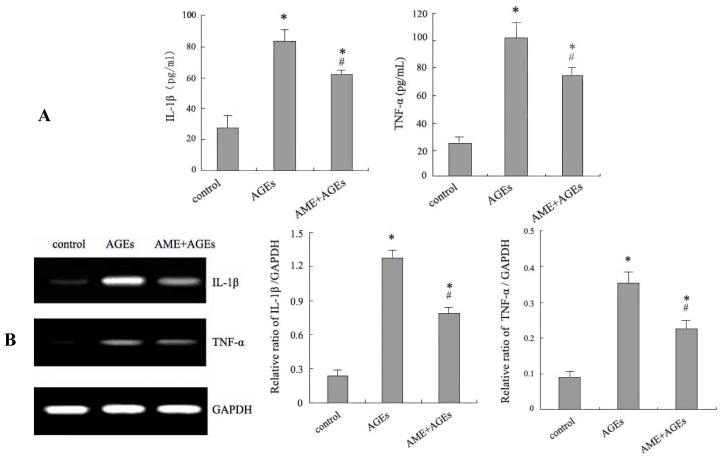
Effects of AGEs and AME on IL-1β and TNF-α production in Ana-1 macrophages. Cells were cultured with AGEs for 24 h with or without pre-treatment with AME for 1 h. The secretion of interleukin-1 beta (IL-1β) and tumor necrosis factor-alpha (TNF-α) was measured by enzyme-linked immunosorbent assay (ELISA) (**A**). The levels of IL-1β and TNF-α mRNA were measured by RT-PCR (**B**). The results represent the mean of six culture wells (mean ± SEM). * *p* < 0.05 compared with control and ^#^ *p* < 0.05 compared with AGEs alone. All of the experiments were performed independently in triplicate.

Treatment of Ana-1 macrophages with 100 mg/L AGEs resulted in significantly increased mRNA levels and secretion of IL-1β and TNF-α (Figure 2A,B). This result suggests that AGEs could increase inflammatory cytokine production by macrophages to trigger an inflammatory response in diabetes.

Subsequently, the function of AME on IL-1β and TNF-α production was examined in AGE-stimulated macrophages. Treatment with AME significantly inhibited the increased mRNA levels and secretion of IL-1β and TNF-α in AGE-stimulated macrophages (Figure 2A,B). These results suggest that AME could suppress the inappropriate inflammatory response induced by AGEs in macrophages. To our knowledge, this study reveals for the first time that *A. membranaceus* could attenuate inflammatory cytokine production in AGE-stimulated macrophages. AME could serve as a potential drug candidate against AGE-induced inflammation in macrophages.

### 2.3. NF-κB and Phospho-p38 MAPK Mediate the Effects of AGEs and AME on Macrophages

Studies have demonstrated that NF-κB activation and MAPK phosphorylation, especially p38 MAPK phosphorylation, are prerequisites of inflammatory cytokine production in stimulated macrophages [21–23]. Moreover, AGEs have been found to increase the phosphorylation of p38 MAPK protein and induce NF-κB activation in macrophages [24].

**Figure 3:**
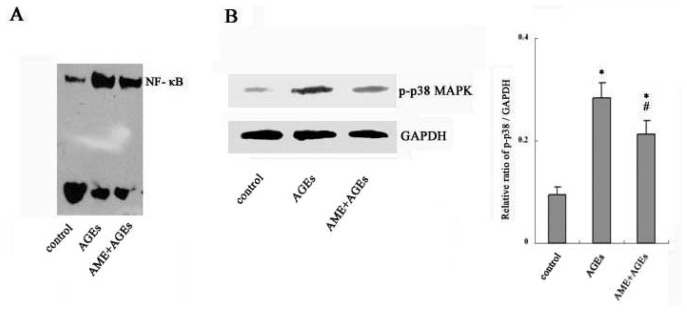
Effects of AME on phosphorylated p38 MAPK (mitogen-activated protein kinase) and nuclear factor (NF)-κB activation in Ana-1 macrophages. Cells were cultured with AGEs for 24 h with or without pre-treatment with AME for 1 h. Nuclear extracts were prepared from cells and analyzed for NF-κB activity by Electrophoretic Mobility Shift Assay (EMSA) (**A**); Phosphorylation of p38 MAPK was determined by western blotting (**B**). The results represent the mean of six culture wells (mean ± SEM). * *p* < 0.05 compared with control and ^#^ *p* < 0.05 compared with AGEs only. All of the experiments were performed independently in triplicate.

Thus, the effects of AME on AGE-induced p38 MAPK phosphorylation and NF-κB activation were examined in macrophages. As shown in Figure 3A,B, treatment of Ana-1 macrophages with AGEs significantly increased phospho-p38 MAPK protein expression and NF-κB activity. Pretreatment with AME markedly attenuated the AGE-induced upregulation of phospho-p38 MAPK protein and NF-κB activity in macrophages.

The results suggest that AME could suppress the AGE-induced activation of p38 MAPK and the NF-κB signaling pathway in macrophages. We propose that the inhibition of p38 MAPK and NF-κB signaling pathways by AME might contribute to attenuating inflammatory cytokine production in AGE-stimulated macrophages.

Furthermore, studies have shown that pathogen-associated molecular patterns induce the activation of proinflammatory macrophages through interaction with distinct metabolic microenvironments and macrophage phenotypes [25]. Thus, we will investigate the functions of AME associated with the interaction of metabolic microenvironments and cell phenotypes in AGE-induced macrophages.

## 3. Experimental Section

### 3.1. Materials

AME was purchased from Chiatai Qingchunbao Pharmaceutical Co. Ltd. (Hangzhou, ZheJiang, China) and formulated according to the standard of the Chinese pharmacopeia. RPMI 1640 and fetal bovine serum (FBS) were purchased from GibcoTM Invitrogen Corporation (Grand Island, NY, USA). Advanced glycation end products (glycoaldehyde-modified AGE-BSA) were purchased from Shanghai Yixin Bio-Technology Co. Ltd. (Shanghai, China). The RevertAid First Strand cDNA Synthesis Kit was purchased from Fermentas International Inc. (Graiciuno, Vilnius, Lithuania). The RNA PCR Kit was purchased from Takara Biotechnology CO. LTD. (Dalian, LiaoNing, China). The NF-κB consensus oligo, SuperECL Plus, and the Nuclear and Cytoplasmic Protein Extraction Kit were obtained from Beyotime Institute of Biotechnology (Haimeng, JiangShu, China). ELISA kits for mouse TNF-α and IL-1β and peroxidase-labeled secondary antibodies were obtained from Wuhan Boster Bio-engineering Limited Company (Wuhan, HuBei, China). MTT was purchased from Sigma-Aldrich (Shanghai, China). The antibody against GAPDH was purchased from Santa Cruz Biotechnology Inc. (Santa Cruz, CA, USA). The anti-phospho-P38 MAPK antibody was purchased from Cell Signaling Technology (Boston, MA, USA).

### 3.2. Preparation of AME

The crude herb and AME were obtained from Chiatai Qingchunbao Pharmaceutical Co. Ltd. and formulated according to the standard of the Chinese pharmacopeia. In brief, the dried roots of AM (1 kg), also known as Radix Astragali or *A. membranaceus* (Fisch.) Bge var. mongholicus Hsiao, were extracted two times using 63% and 86% ethanol. Then, ethanol was removed. The prepared total AM was dissolved in deionized water at a ratio of 1 mL:2.5 g and filtered. The filtrate was boiled for 5 min, and deionized water was added to 1000 mL. The AM extract was filtered and pasteurized.

### 3.3. Cell Culture

The Ana-1 murine macrophage cell line was obtained from the cell bank of Shanghai Institutes for Biological Sciences, Chinese Academy of Sciences (Shanghai, China). Cells were maintained in RPMI 1640 medium supplemented with 10% fetal bovine serum, 100 units/mL penicillin and 100 μg/mL streptomycin and incubated at 37 °C in 5% CO_2_ humidified air.

### 3.4. Assessment of Cell Viability

Macrophages (100 μL) were seeded at a density of 5 × 10^4^ cells/mL and incubated with AME (5–40 μL/mL) or AGEs (25–200 mg/L) in 96-well plates. After 24 h of incubation, MTT solution was added to each well for 4 h. Finally, the blue salt in each well was dissolved, and the plates were read by a microplate reader with RPMI 1640 as a blank and cell culture medium as a control. We determined the concentration of AME (40 μL/mL) and AGEs (100 mg/L) for the subsequent experiments based on the cell viability results.

### 3.5. Assessment of TNF-α and IL-1β Secretion

Ana-1 macrophages were plated at 5 × 10^5^ cells/well in 24-well plates overnight. Macrophages were cultured with AGEs for 24 h or pre-treated with AME for 1 h before culture with AGEs. The levels of TNF-α and IL-1β in culture supernatants were determined using commercially available enzyme-linked immunosorbent assay (ELISA) kits according to the manufacturer’s instructions.

### 3.6. Detection of mRNAs by RT-PCR

In total, 1 × 10^6^ Ana-1 macrophages were plated in a 6-well culture plate, and AGEs were added for 24 h with or without AME pre-treatment for 1 h. Equal amounts of RNA were isolated using TRZOL Reagent and reverse transcribed into cDNA using the RevertAid First Strand cDNA Synthesis Kit according to the protocol described by the manufacturer. Products were amplified from cDNA templates with 35 cycles using primers for TNF-α and IL-1β (TNF-α forward: AAATTCGAGTGACAAGCCTGTAG and reverse: GAGAACCTGGGAGTAGACAAGGT; IL-1β forward: CAAGTGTCTGAAGCAGCTATGG and reverse: GAGATTTGAAGCTGGATGCTCT). The amount of TNF-α and IL-1β was determined and normalized to the amount of GAPDH cDNA.

### 3.7. Western Blot Analysis

In total, 1 × 10^6^ Ana-1 macrophages were plated in a 6-well culture plate, and AGEs were added for 24 h with or without AME pre-treatment for 1 h. The cells were lysed with dissociation solution containing phosphatase, protease inhibitors and PMSF. The protein extracted from macrophages was electrophoresed on SDS-polyacrylamide gels (10%) and transferred to nitrocellulose membranes. Membranes were incubated with blocking buffer and then incubated with anti-phospho-p38 MAPK antibody at 4 °C overnight. The membranes were incubated with a peroxidase-conjugated secondary antibody and visualized by a super-enhanced chemiluminescence detection system. Densitometric analysis was performed by normalizing the band density to GAPDH.

### 3.8. Electrophoretic Mobility Shift Assay (EMSA)

In total, 1 × 10^6^ Ana-1 macrophages were treated with AGEs for 24 h with or without AME pre-treatment for 1 h. The nuclear extract of cells was mixed with binding buffer and nuclease-free water. Then, biotin-labeled oligonucleotide probes of NF-κB were added according to the manufacturer’s protocol. The mixture was subjected to electrophoresis on 6% polyacrylamide gels and transferred to a nylon membrane. A chemiluminescent detection method was used according to the manufacturer’s description, and membranes were exposed to X-ray films.

### 3.9. Statistical Analysis

The data are expressed as the mean ± SEM. Statistical analysis was performed by SPSS software using a one-way analysis of variance (ANOVA). Differences with a *p* value less than 0.05 were considered to be statistically significant.

## 4. Conclusions

Collectively, the results suggest that AME can inhibit the AGE-induced production of inflammatory cytokines in macrophages to down-regulate inflammation through p38 MAPK and NF-κB signaling pathways, indicating its potential application as an immunoregulatory agent in diabetes. Further studies will determine the functions and appropriate concentration of AME against inflammatory reactions in diabetic rats.
